# Severe Acute Malnutrition among Unoperated Ethiopian Children with Congenital Heart Disease: A Wake-up Call to Reverse the Situation, A Retrospective Cross-Sectional Study

**DOI:** 10.4314/ejhs.v30i5.9

**Published:** 2020-09

**Authors:** Bruk Assefa, Henok Tadele

**Affiliations:** 1 Department of Pediatrics and Child Health, College of Medicine and Health Sciences, Hawassa University; 2 Department of Pediatrics and Child Health, College of Health Sciences, Addis Ababa University

**Keywords:** Congenital heart disease, severe acute malnutrition, Ethiopia

## Abstract

**Background:**

Children with Congenital Heart Disease (CHD) are at increased risk for severe acute malnutrition (SAM). We aimed to determine the magnitude and determinants of SAM among children with CHD in a tertiary hospital.

**Methods:**

Retrospective cross-sectional study was conducted among children with CHD between 2016 and 2019. Clinical and anthropometric data were retrieved from medical records. Anthropometric assessment was done by using WHO standard growth curves. Data analysis was done using Statistical Package for Social Sciences V22. Statistical significance was set at p-value <0.05, and multivariable logistic regression was used to determine predictors.

**Results:**

There were 2400 pediatric admissions during the study period, CHD accounted for 6.5%(156) of admissions. For review, 141 records were eligible. The gender distribution was comparable, males 51.1% (72). Infants (<12 months) and older children (≥12 months) accounted for 57.4% (81) and 42.6% (60) of study subjects, respectively. SAM was documented in 51.8% (73) of the study subjects, [95% CI: 44.7–60.2]. Infants had higher odds of SAM compared to children aged ≥12 months[adjusted odds ratio (AOR)= 4.48, 95%CI:2.07–9.70]. Anemic children had higher odds for SAM[AOR =3.76, 95% CI:1.54–9.18]. Children without acyanotic CHD with heart failure(HF) were 58% less likely to develop SAM[AOR= 0.42, 95% CI:0.19–0.96].

**Conclusion:**

The burden of SAM among children with CHD is high. Younger age, anemia and acyanotic CHD with HF predicted SAM. Screening for anemia and targeted anthropometric assessment are recommended for early SAM detection.

## Introduction

Double burden of malnutrition with childhood undernutrition and adult obesity is documented in low and middle-income countries (1,2). Severe Acute Malnutrition (SAM) is considered when weight for height(WFH) is <-3 Z score, bilateral pitting edema or low mid upper arm circumference (MUAC) is documented(3). Malnutrition is a common problem among children with Congenital Heart Disease (CHD)(4). Stunting and wasting are reported in 37% and 7% of Ethiopian children under the age of five, respectively(5).

Several mechanisms are proposed for growth failure among children with CHD. Decreased intake, increased energy requirements and presence of pulmonary hypertension are the main factors leading to malnutrition. Decreased intake could be related to anorexia following the chronic hypoxemia, and several other immune and neurohormonal disturbances are also postulated(6–10). Early and timely CHD interventions are shown to improve the catch-up growth(11–13).

Malnutrition in CHD patients can be categorized into different groups depending on the presence or absence of cyanosis/pulmonary hypertension/heart failure. Several combinations including acyanotic patients with pulmonary hypertension (patients with left-to-right shunt and pulmonary hypertension), acyanotic patients without pulmonary hypertension and cyanotic patients are used(4, 14–16).

SAM was documented among 31–71.4% of children with CHD. Presence of anemia, age at presentation, low arterial oxygen saturation, heart failure, poor dietary history, pulmonary hypertension and lack of surgical interventions predicted SAM(4, 17–20). SAM is managed using the World Health Organization's (WHOs) treatment protocol(21). Preoperative nutritional care is highly recommended to improve the postoperative outcome of CHD (12). Treatment of CHD in most parts of Africa, including Ethiopia, is provided by overseas voluntary mission or humanitarian aid, and the majority of the patients do not have access to the service. Success of such treatment models in sustaining the care has been questioned, and reconsideration was suggested(22,23). Global call for coordination of such pediatric cardiac surgery mission efforts was also recommended(24). Evidence on CHD related SAM is scarce from Africa and absent from Ethiopia. We aimed to assess the magnitude and predictors of SAM among unoperated pediatric CHD.

## Methods

**Setting**: The study was conducted at Hawassa University Comprehensive Specialized Hospital (HUCSH), Hawassa, 271km South of Addis Ababa, Ethiopia's capital. HUCSH is the first referral and teaching hospital in Southern Ethiopia. It serves a catchment population of over 18 million. It is a referral hospital for Southern Ethiopia and its neighboring regional states of Ethiopia. Pediatrics Department provides services for outpatient, emergency and admitted cases. There is an established therapeutic ward for care of children with SAM. SAM patients receive intensive care and followup by trained and dedicated nurses, intern and resident doctors, and pediatric consultant physicians. Admitted workup and treatment of CHD cases also include radiologic studies, echocardiographic imaging and laboratory tests.

**Study design**: Retrospective cross-sectional study on the medical records of CHD patients seen at HUCSH between 2016 and 2019

**Study population**: All pediatric patients, aged less than 14 years, with CHD who had received admitted care at HUCSH

**Inclusion criteria**: All children between 2months and 14 years of age with echocardiographic documented CHD

**Exclusion criteria**: Incomplete charts, children with comorbid condition like developmental delay, Downs Syndrome and other genetic abnormalities

**Operational definitions**: Moderate malnutrition was defined when weight for height (WFH), weight for age (WFA), and height for age (HFA) are between -3 and -2 Z-scores on WHO standard growth curves. On the other hand, SAM was considered when WFH was <-3 Z score, bilateral pitting edema or low mid upper arm circumference (MUAC) for age(3). Underweight and stunting were defined when the weight for age and height for age were < -2 Z scores, respectively. Wasting and severe wasting were considered when the weight for height was < -2 Z score and < -3 Z scores, respectively(25). Anemia was defined when hemoglobin concentration was <11g/dl, <11.5g/dl, and <12g/dl in under five years, 5–11 years and 12–14 years of age, respectively(26). Cardiomegaly was defined when cardiothoracic ratio was > 60% in neonates, and >55% in older children (27). Acyanotic CHD was defined as anatomic connection between the pulmonary and systemic circulations with left to right shunts (28). Cyanotic CHD was defined as heart lesions resulting in cyanosis owing to right sided heart obstructive lesions, left heart obstructive lesions, and mixing lesions(29). Infants are children below the age of one year(30).

Pulmonary hypertension (PH) was considered when the peak tricuspid regurgitation velocity was >2.8m/sec and other parameters like blunted inferior vena diameter respiratory cycle variation were present (31). Patients were grouped into different classes: group I, acyanotic CHD without heart failure or pulmonary hypertension; group II, acyanotic with heart failure; group III, acyanotic CHD with pulmonary hypertension; group IV, cyanotic CHD(16).SAM treatment outcome was assessed on hospital discharge and was defined as death or survival.

**Sample size**: A sample size of 114 was calculated using single population proportion formula with assumptions of 92% malnutrition proportion among CHD, 95% confidence interval with 5% margins of error (4).A total of 156 consecutive medical records were retrieved during study period from medical records archive, and fifteen were excluded due to incomplete information and documentation of syndromic or genetic abnormality diagnosis. Finally, one hundred forty-one medical records fulfilling the predefined criteria were selected and included in the study.

**Study variables**: The dependent variable was severe acute malnutrition. Age, sex, income, CHD type, pulmonary hypertension, nutritional history and heart failure were the independent variables.

**Data collection**: Structured questionnaire was prepared and data were collected from patient medical records. Socio-demographic data, anthropometric data, clinical, echocardiography results and summary of important laboratory findings were retrieved from the medical records of patients.

**Data analysis**: The collected data were analyzed using descriptive and inferential statistics. Statistical package for social sciences (SPSS) version 22 software was used to analyze the data. Frequencies and percentages were used to present categorical data. Parametric tests were used to assess the association between variables and outcome. Binary logistic regression was used to check the association between dependent and independent variables. Variables with Pvalue < 0.25 on binary logistic regression were used for multivariable logistic regression for controlling the possible effect of confounders. Finally, variables which had an independent association with SAM were identified on the basis of adjusted odds ratio(AOR), 95% CI and P-value < 0.05.

**Ethical approval**: Ethical approval and waiver of consent for the study was obtained from the Institutional Review Board of Hawassa University.

## Results

**Socio-demographic profile**: There were 2400 pediatric admissions during the study period, and CHD accounted for 6.5% (156) of the admissions. One hundred forty-one medical records were eligible for review and included in the study. The gender distribution was comparable, males 51.1% (72) of study subjects. Most of the study subjects were from the Southern regional state of Ethiopia, 55.3% (78), while the remaining ones were from the neighboring Oromia regional state of Ethiopia, 44.7% (63). Infants (2–12 months) accounted for 57.4% (81) of the study subjects.

**Anthropometric data**: Underweight and moderate malnutrition by MUAC were documented in 70(49.6%) and 34(24.1%) of the study subjects, respectively. Stunting was reported in 42(29.8%) of the study subjects. SAM was reported in 53(37.6%) and 39(27.7%) of the study subjects using MUAC and WFH, respectively. SAM using both MUAC and WFH was documented in 73(51.8%) of the study subjects (Table 1).

**Table 1:** Anthropometric Measurements of Children with Congenital Heart Disease at Hawassa University Hospital, Hawassa, 2016–2019

Anthropometric parameter	Frequency	Percent
Weight for age		
Normal	60	42.6
Underweight	70	49.6
Height for age		
Normal	99	70.2
Stunted	42	29.8
MUAC^$^ for age		
Severe malnutrition	53	37.6
Moderate malnutrition	34	24.1
Mild malnutrition	11	7.8
Normal	43	30.5
Weight for height		
Normal	52	36.9
Wasting	89	63
Severe wasting (by Weight for height)		
Yes	39	27.7
No	102	72.3
^$^SAM (WFH and MUAC*)		
Yes	73	51.8
No	68	48.2

&MUAC-mid upper arm circumference, $SAM-severe acute malnutrition, *WFH weight for height

**Clinical data**: Pulmonary hypertension was documented in 26(18.4%) of CHD, and moderate severity predominates, 12(46.1%). cardiomegaly and anemia were reported in 112(79.4%) and 39(27.7%) of the study subjects, respectively. The majority of the study subjects had been survived up on discharge, 123(87.2%). Most of the study subjects had acyanotic CHD with heart failure, 90(63.8%) (Table 2). The common types of CHD included ventricular septal defect, patent ductus arteriosus, tetralogy of fallot, endocardial cushion defect and atrial septal defects contributing to 79(56%), 16(11.3%), 15(10.6%), 13(9.2%) and 11(7.8%) of the study subjects, respectively. None of our study subjects had undergone surgical or definitive intervention for CHD.

**Table 2:** Clinical Characteristics of Malnutrition among Children with Congenital Heart Disease at Hawassa University Hospital, Hawassa, 2016–2019

Variable	Category	Frequency (N=141)	Percent
Pulmonary Hypertension	Yes	26	18.4
	No	115	81.6
Severity of Pulmonary Hypertension	Mild	5	19.2
	Moderate	12	46.1
	Severe	9	34.6
Cardiomegaly	Yes	112	79.4
	No	29	20.6
Pulmonary edema	Yes	49	34.8
	No	92	65.2
Anemia	Yes	39	27.7
	No	102	72.3
Acyanotic CHD^%^ without ^&^HF/PHTN*	Yes	16	11.3
	No	125	88.7
Acyanotic CHD with HF	Yes	90	63.8
	No	51	36.2
Acyanotic CHD with PHTN	Yes	23	16.3
	No	118	83.7
Cyanotic CHD without PHTN/HF	Yes	26	18.4
	No	115	81.6
Treatment outcome at discharge	Survived	123	87.2
	Died	18	12.8

^%^CHD-congenital heart disease, ^&^HF-heart failure, * PHTN-pulmonary hypertension

**Magnitude and determinants of malnutrition**: The overall magnitude of SAM in this study was 51.8%(73).

**Bivariate analysis**: From all variables analyzed, seven variables (age, sex, anemia, pulmonary hypertension, cardiomegaly, pulmonary edema, and acyanotic CHD with heart failure) were associated with SAM on bivariate analysis (Table 3). Variables with p-value < 0.25 were selected for multivariable analysis

**Table 3:** Results of Bivariate Analysis in Children with Congenital Heart Disease at Hawassa University Hospital, Hawassa, 2016–2019

Variable	Category	SAM^#^		^$^COR (95% %CI)	P. value
		Yes	No		
Age	≥12 months	20	40	1	0.000
	2–12 months	53	28	7.95(2.70–23.35)	
Anemia	Yes	44	58	1	0.001
	No	29	10	3.82(1.68–8.66)	
Cardiomegaly	Yes	61	51	1	0.21
	No	12	17	0.59(0.59–2.37)	
Acyanotic CHD^^^ with ^&^HF	Yes	19	32	1	0.05
	No	41	49	0.49(0.24–1.04)	
	Yes	16	10	1.62(0.68–3.88)	0.27
Pulmonary *HTN	No	57	58	1	
	Male	40	32	1.36(0.70–2.64)	0.35
Sex	Female	33	36	1	
	Yes	24	25	0.84(0.42–1.68)	0.62
Pulmonary edema	No	49	43	1	

^CHD-congenital heart disease, ^&^HF-heart failure, *HTN-hypertension,^#^SAM-severe acute malnutrition, ^$^COR-crude odds ratio, ^%^CI- confidence interval

**Multivariable analysis result**: Infants (2–12 months) had four times higher odds of SAM compared to children aged 12 months and above[AOR =4.48, 95% CI: 2.07–9.70]. Those who had anemia were four times more likely to develop SAM compared to their counterparts, [AOR =3.76, 95% CI:1.54–9.18] whereas those who had no acyanotic CHD with HF were 58% less likely to have SAM compared to their counterparts [AOR =0.42, 95% CI: 0.19–0.96] (Table 4).

**Table 4:** Factors Associated with Severe Acute Malnutrition among Children with CHD at Hawassa University Hospital, Hawassa, 2016–2019

Variable	Category	SAM		AOR (95% CI)	P. value
				
		Yes	No		
Age	≥12 months	20	40	1	0.000
	2–12 months	53	28	4.48(2.07–9.70) *	
Anemia	Yes	44	58	1	0.004
	No	29	10	3.76(1.54–9.18) *	
Cardiomegaly	Yes	61	51	1	0.2
	No	12	17	1.86(0.71–4.87)	
Acyanotic CHD with HF	Yes	19	32	1	0.04
	No	41	49	0.42(0.19–0.96) *	

* Significant p-value <0.05, COR = Crude Odds Ratio, CI = Confidence interval, AOR = Adjusted Odds Ratio

## Discussion

The magnitude of severe acute malnutrition among CHD in this study was 51.8%, 95% CI (44.7%–60.2%). This magnitude is lower than studies conducted in Egypt (71.4%) and Nigeria (61.2%). This difference could be due to the age of inclusion, settings and capacity of the institutions and design differences (17,18). Our report is higher than reports from Uganda(31.5%) and Iran (39.8%)(15,19). The higher figure in our study could be a reflection of the high national burden of malnutrition in Ethiopia(5). In the current study, different forms of malnutrition were documented. Underweight, stunting, and moderate and severe wasting were documented in 70(49.6%), 42(29.8%), 34(24.1%) and 16(11.1%) of the study subjects, respectively. Comparable results were reported from Uganda and Nigeria; undernutrition assessment in a tertiary hospital in Tanzania documented a similar pattern (18,19,32).A higher figure was reported from Iraq with underweight and wasting contributing to 32% and 65% of CHD(16). Nevertheless, this study only included infants as study subjects.

In this study, the majority of our study subjects had acyanotic congenital heart disease with heart failure. This is comparable to a previous report from the same hospital and a study done in Addis Ababa, Ethiopia(33,34). Heart failure is documented to result in energy imbalance in cardiac patients by increasing basal metabolic rate due to inflammation or chronic disease, and hence resulting in malnutrition(6, 7, 35). Additionally, children who had no acyanotic CHD with HF were 58% less likely to develop SAM compared to their counterparts. This is opposed to the evidence that cyanotic patients are at higher risk of growth failure due to prolonged hypoxemia(35,36). However, our report should be seen in light of the small size of cyanotic heart disease children in this study as the more severe cyanotic patients might have died early due to the natural course of the illness and/or lack of interventions.

In the current study, infants (2–12 months) were four times more likely to develop SAM compared to children of 12 months and above. This is consistent with reports from Nigeria and India(4,13). Infancy is linked with a higher energy demand for accelerated growth, and it is also the time where the risk for chest infection is very high owing to CHD related left to right shunts (35,38,39).

In this study, children who had anemia were four times more likely to develop SAM compared to their counterparts. This finding is consistent with reports from Nigeria and Uganda(18,19). Anemia could be a marker of underfeeding especially in children with CHD owing to the feeding challenges among them (35,38). Moreover, it is documented that anemia among children with severe acute malnutrition is of nutritional origin even demanding transfusion (40).

Our study has limitations. It is a retrospective and single center study, and several other determinant factors including intake and feeding pattern which assist in determining the primary nutritional issues were not assessed. It was also not possible to validate the documented anthropometric measurements. However, our study reports the magnitude and determinant factors for SAM among children with the non-intervened CHD in the Ethiopian setting. It also calls for timely attention to reverse the situation and to avert the productive loss in the long run.

In conclusion, a high severe acute malnutrition burden among children with congenital heart disease is documented. Young age, anemia and acyanotic CHD with HF were factors positively associated with severe acute malnutrition. Screening for anemia and targeted anthropometric assessment are recommended for better and early detection of SAM.
